# Ocular Side Effects of Sildenafil That Persist Beyond 24 h—A Case Series

**DOI:** 10.3389/fneur.2020.00067

**Published:** 2020-02-07

**Authors:** Cüneyt Karaarslan

**Affiliations:** World Eye Hospital, Adana, Turkey

**Keywords:** headache, heartburn, side effects, sildenafil citrate, visual disturbance

## Abstract

Acute secondary effects of sildenafil, a first-line pharmacotherapy for erectile dysfunction (ED), include headache, heartburn, skin flush, and vision changes. Generally, these effects subside within 5 h. This is a retrospective report of 17 cases in which patients experienced visual disturbances following 100-mg sildenafil use that persisted for more than 24 h. All 17 patients were healthy men taking sildenafil for the first time without prescriptions who sought consultation at our clinic within 48 h of taking the drug. Diagnostic tests indicated that out of the 17 patients, nine had photophobia, 13 had disrupted color perception, nine had impaired visual acuity, three had deficiencies in stereopsis, six had disrupted contrast sensitivity, and eight had abnormally dilated pupils. These disturbances resolved within 21 days in all 17 cases. There was near-full case overlap between photophobia and color vision impairment. In conclusion, because some individuals have heightened sensitivity to sildenafil, perhaps due to metabolic variance, patients should be started on a modest trial dose.

## Introduction

Sildenafil (UK-92,480, a.k.a., Viagra^®^, Pfizer Inc. New York, NY), originally a putative pharmacotherapy for hypertension, has become a first-line treatment for erectile dysfunction [ED; (1)]. Pharmacologically, sildenafil prevents the degradation of cyclic guanosine monophosphate (cGMP) by inhibiting the activity of cGMP-specific phosphodiesterases (PDEs). The resultant increases in cGMP levels increase activation of cGMP-dependent protein kinase, leading to vasodilation and cavernosal smooth muscle relaxation, thereby facilitating penile erection upon sexual stimulation (2). Oral sildenafil is absorbed rapidly, with blood levels peaking 0.5–1.5 h after it is consumed, and then metabolized in the liver with a metabolic half-life of 3–5 h (3).

Sildenafil is considered to be generally safe. In a study of 15 patients using 50 mg sildenafil twice weekly for 3 months, no significant effects on best-corrected visual acuity or introaocular pressure were observed (4, 5). Acute secondary effects of sildenafil include headache, heartburn, skin flush, and vision changes such as blurred vision, photophobia, and cyanopsia [i.e., blue-tinted vision; (6)]. Here, we report a case series of patients who experienced visual disturbances that persisted for more than 24 h in response to taking sildenafil.

## Case Series

A series of 17 cases was included in this retrospective report. The patients were all adult men with a mean (±standard deviation) age of 47.0 ± 5.8 years (range: 38–57 years). All 17 patients had taken a single 100-mg sildenafil pill 24–48 h before seeking consultation at our clinic between August of 2017 and March of 2019. In all cases, the purpose of taking sildenafil was to improve the patient's ability to achieve and maintain an erection. All 17 patients were trying sildenafil for the first time and taking the medication without prescriptions. Also, all patients were new admissions who had not received any previous ocular examinations in the clinic and had never reported ocular or visual problems mimicking sildenafil-related signs and symptoms. All patients were evaluated after 10 and 21 days.

During the consultation at our clinic, each patient underwent the following evaluations: a Snellen chart test of visual acuity, a cone contrast test of color vision, a stereo butterfly test of depth perception (Keeler, USA), a Pelli Robson test of contrast sensitivity, an Orbscan^®^ corneal topographic assessment, and a pupil diameter measurement. Regarding the color vision assessment, we adopted the manufacturer's interpretation rubric, which is as follows: scores ≥90 are considered normal, scores in the range of 75–90 indicate a potential acquired impairment, and scores <75 indicate a definite impairment. Pelli Robson test results were interpreted as follows: 2.0 = normal; 1.0–2.0 = impaired; and < 1.0 = disability. The patients' pupils were measured with the aforementioned Orbscan^®^ device in dim light without any contracting or dilating stimulation; diameters in the range of 2.0–4.0 mm were considered normal.

The following acute secondary effects of sildenafil were reported by the patients: headache (*n* = 13), heartburn (*n* = 11), blurred vision (*n* = 8), and cyanopsia (*n* = 12). Regarding the symptom of cyanopsia, the affected patients reported very intensely blue- colored vision with red-green color blindness (i.e., reds and greens appeared to be brownish hues). None of the 17 patents had a history of ocular pathology (including glaucoma) or any diagnosed systemic disease.

The individual patients' results for the aforementioned tests are reported in Table 1. Briefly, 9/17 patients (52.9%) exhibited some degree of clinical photophobia, including five mild presentations, two moderate, one severe, and one very severe presentation. The color vision assessment indicated that 13/17 patients (76.5%) had at least one score (for S, L, and/or M cones) that did not reach the 90-point normal threshold; five of these patients each had at least one score indicative of a definite impairment, and the remaining eight patients had 1–3 scores in the potential acquired impairment range. None of the 17 patients were innately color blind. Regarding visual acuity, eight patients had 20/20 vision without optical correction. The remaining nine patients, who all achieved a best corrected visual acuity of 20/20, complained of impaired vision relative to their normal visual acuities. On the day of the examination, their uncorrected visual acuities ranged from 10/20 to 18/20. Stereo butterfly testing showed that 3/17 patients (17.6%) had a deficiency in stereopsis; these patients retained a sense of depth perception within objects but lacked their normal full stereopsis abilities to sense their distances from objects and distances between objects in the environment. Pelli Robson contrast sensitivity testing indicated that 6/17 patients (35.3%) had a transient contrast sensitivity impairment (*n* = 5) or disability (*n* = 1). Pupil diameter measurements indicated that 8/17 patients (47.1%) had abnormally dilated pupils (>4.0 mm). Pupillary reactions to light were tested, and no relative afferent defects were found in any of the patients. In all cases, the patients had symmetrical pupillary dilations.

**Table 1:** Summary of eye examination findings by individual patients.

**Case**	**Age, years**	**Photo-phobia**	**Color vision, S/L/M**	**Visual acuity**	**Stereopsis/depth perc**.	**Contrast sensitivity**	**Pupil diam, mm**
1	38	+ + + +	100/60/70^**^	20/20	Stereoscopic	1.5	3.5
2	39	+ + +	100/70/70^**^	20/20	Stereoscopic	1.5	4.0
3	40	++	100/70/70^**^	20/20	Stereoscopic	2.0	4.0
4	42	++	90/90/80^*^	18/20	Stereoscopic	2.0	3.9
5	42	+	90/90/90	20/20	Stereoscopic	2.0	4.4
6	43	−	90/90/90	12/20	Stereoscopic	1.5	5.1
7	44	−	90/100/90	20/20	Stereoscopic	2.0	4.5
8	45	−	80/90/90^*^	10/20	Stereoscopic	2.0	4.2
9	46	+	100/80/90^*^	18/20	Stereoscopic	2.0	4.3
10	48	−	90/100/90	20/20	Stereoscopic	2.0	4.6
11	49	+	80/90/90^*^	18/20	Depth perc. only	1.0	4.1
12	51	−	80/80/80^*^	18/20	Depth perc. only	1.5	4.2
13	53	+	80/80/80^*^	20/20	Stereoscopic	2.0	4.0
14	53	−	80/90/100	10/20	Depth perc. only	1.5	3.5
15	54	−	70/90/90^**^	12/20	Stereoscopic	2.0	3.5
16	55	+	90/80/90^*^	20/20	Stereoscopic	2.0	4.0
17	57	−	90/90/90	18/20	Stereoscopic	2.0	4.1

*Photophobia: ++++, very severe; +++, severe; ++, moderate; and +, mild. S, S cones; L, L cones; M, M cones; diam, diameter; and perc., perception*.

* *Potential impairment*;

** *Definite impairment*.

The patients were advised that their visual disturbances should resolve spontaneously within 10 days. As a result of a final ocular examination at 21 days, it was confirmed that the patients' visual disturbances had, in fact, resolved in all 17 cases; this revealed that all ocular effects were transient.

## Discussion

In this report, we describe 17 cases of men in generally good health who experienced vision changes that persisted for at least 24 h after taking sildenafil. The persistence of these symptoms was concerning to the patients given that the effects of sildenafil were expected to last for only 3–5 h. Notably, there was strong overlap between clinically detectable photophobia and color vision impairment, with 8/9 patients with transient photophobia having also exhibited color vision impairment and 9/11 patients with transient color vision impairment having exhibited photophobia.

It is possible that the extended durations of our patients' visual secondary effects of sildenafil were related to the fact that they all had taken the maximum recommended therapeutic dose for ED, 100 mg, despite the recommended starting dose for sildenafil, which is 50 mg with the option to decrease to 25 mg or increase up to 100 mg depending upon the patient's reaction to the drug. Although temporary mild color discrimination impairment is often experienced within a few hours of taking sildenafil, coinciding with a period of maximal sildenafil levels in the patient's blood circulation (3), there is no consistent pattern of long-term ocular effects of sildenafil. Sildenafil is considered to be generally safe, given that (1) it has not shown any long-term adverse effects on eye structure or function in high-therapeutic-dose toxicology studies in animals, and (2) it has been shown to have no effects on visual acuity, visual field, or contrast sensitivity in human subjects with opthalmic diseases (7).

Recently, Rosen et al. (8) reported the case of a 57-year-old man who, upon taking a single 100-mg dose of sildenafil under the direction of his urologist in preparation for a radical prostatectomy, experienced a sensation of unusual brightness of incoming visual stimulation combined with abnormal color vision that persisted beyond 5 h. These effects had fully resolved within 7 days after discontinuing sildenafil. He was switched to tadalafil (Cialis^®^), another PDE inhibitor. However, when his tadalafil dose was increased from 5 mg (which was ineffective for urologic improvement and without secondary effects) to 20 mg, his visual disturbance symptoms returned within a few hours, resolving completely within 14 days after ceasing tadalafil use. The experience of Rosen et al.'s (8) patient appears to have been consistent with the experiences of many of the patients in the present report. Such cases suggest that a relatively small subpopulation of people are at risk of disturbingly intense secondary effects of PDE inhibitors and thus support the practice of starting patients on a modest dose when prescribed a PDE inhibitor.

The efficacy of sildenafil for ED has been attributed principally to its actions on PDE5 in vascular tissues of the corpura cavernosa (3). However, its transient effects on vision, especially cyanopsia attributed to rod sensitization, are likely mediated by PDE6, which is expressed specifically in rod and cone photoreceptor cells in the retina and shows minor inhibition in response to sildenafil (3). Dundar et al. (5) described the temporary effects of sildenafil on ocular hemodynamic effects. These effects included elevated peak systolic velocity, mean velocity, and end-diastolic velocity of the opthalmic arteries, detectable 1 h after sildenafil ingestion in the absence of any significant hemodynamic changes in the central retinal artery or short posterior ciliary artery and without significant changes in intraocular pressure, color vision, visual acuity, or systemic blood pressure (4). The marked alterations in vision induced by sildenafil in the present case series and induced by tadalafil in the aforementioned case reported by Rosen et al. (8) extend in time and severity beyond the well-documented transient cyanopsia that is commonly experienced under the acute influence of a PDE inhibitor. Of note, anterior ischemic neuropathy has been reported in association with PDE-5 inhibitor (e.g., sildenafil, vardenafil, and tadalafil) use. Relative afferent pupillary defect, decreased color vision, and visual field defect indicate this type of neuropathy (9, 10). Recently, Zahavi et al. (11) demonstrated that sildenafil may induce ischemic retinal changes in a mouse model. The mouse model showed optic nerve stroke (NAION) induced by high dose administration of sildenafil, followed by retinal ganglion cell loss. On the other hand, mice that underwent optic nerve crush had shown a neuroprotective effect following sildenafil injection.

Currently, the biological factors that make particular individuals susceptible to exceptionally intense visual disturbances in response to PDE inhibitors (e.g., sildenafil) are not known. It is possible that this vulnerability may be related to individual differences in the metabolism and clearance of sildenafil. Sildenafil is metabolized primarily by the cytochrome P450 3A4 isoenzyme in the liver (3). Thus, it would be of interest to determine whether individuals who exhibit this sensitivity are carriers of a genetic variant of the CYP3A4 gene or a gene encoding a related molecule. It would be of interest to determine whether such sensitive individuals are also unusually sensitive to the effects other drugs and toxins metabolized by the cytochrome P450 3A4 isoenzyme. It is also notable that the patients with the most pronounced photophobia and color vision disturbance symptoms were the youngest patients in the series. Thus, it would be of interest to determine whether age is a significant determining factor in sildenafil sensitivity. The limitation of the present study was that it was retrospective in nature. Therefore, there is no available baseline data concerning the patients prior to sildenafil intake.

In conclusion, all patients' ocular side effects spontaneously resolved. The present case series highlights the need for awareness of a relatively rare heightened sensitivity to PDE inhibitors such as sildenafil. These findings support the practice of starting patients on a modest dose of sildenafil for ED and other indications. Also, these findings further support the notion that a 100-mg dose of sildenafil should be reserved for patients who did not obtain satisfactory results with a 50-mg trial dose and did not experience heightened sensitivity to the drug as a result of the trial dose.

## Data Availability Statement

The datasets generated for this study are available on request to the corresponding author.

## Ethics Statement

The studies involving human participants were reviewed and approved by World Eye Hospital Ethics Committee. The patients/participants provided their written informed consent to participate in this study.

## Author Contributions

The author confirms being the sole contributor of this work and has approved it for publication.

### Conflict of Interest

The author declares that the research was conducted in the absence of any commercial or financial relationships that could be construed as a potential conflict of interest.
